# Analysis of bearing wear, whole blood and synovial fluid metal ion concentrations and histopathological findings in patients with failed ASR hip resurfacings

**DOI:** 10.1186/s12891-017-1894-5

**Published:** 2017-12-11

**Authors:** Lari Lehtovirta, Aleksi Reito, Jyrki Parkkinen, Harry Hothi, Johann Henckel, Alister Hart, Antti Eskelinen

**Affiliations:** 1 Faculty of Medicine, University of Tampere, Tampere, Finland; 2 Coxa Hospital for Joint Replacement, Tampere, Finland; 3 Fimlab Laboratories Oy, Tampere, Finland; 4 University College London, London, UK

**Keywords:** Metal-on-metal hip replacement, Adverse reaction to metal debris, ARMD, ALTR, ALVAL, Wear, Histopathology

## Abstract

**Background:**

Adverse Reaction to Metal Debris (ARMD) is still a major reason for revision surgeries in patients with metal-on-metal (MoM) hip replacements. ARMD consists of a wide range of alterations in periprosthetic tissues, most important of which are metallosis, inflammation, pseudotumors and necrosis. Studies investigating histopathological findings and their association to implant wear or indirect measures of wear have yielded inconsistent results. Therefore, we aimed to investigate bearing surface wear volume, whole blood and synovial fluid metal ion concentrations, histopathological findings in periprosthetic tissues and their associations.

**Methods:**

Seventy-eight patients with 85 hips revised for ARMD were included in the study. Prior to revision surgery, all patients had whole blood chromium and cobalt ion levels assessed. In revision surgery, a synovial fluid sample was taken and analyzed for chromium and cobalt. Periprosthetic tissue samples were taken and analyzed for histopathological findings. Explanted implants were analyzed for bearing wear volume of both acetabular cup and femoral head components.

**Results:**

Volumetric wear of the failed components was highly variable. The total wear volume of the head and cup had a strong correlation with whole blood chromium and cobalt ion concentrations (Cr: ρ = 0.80, *p* < 0.001 and Co: ρ = 0.84, *p* < 0.001) and a bit weaker correlation with fluid chromium and cobalt ion concentrations (Cr: ρ = 0.50, *p* < 0.01 and Co: ρ = 0.41, *p* = 0.027). Most tissues displayed only low-to-moderate amounts of macrophages and lymphocytes. Total wear volume correlated with macrophage sheet thickness (ρ = 0.25, *p* = 0.020) and necrosis (ρ = 0.35, *p* < 0.01). Whole blood chromium and cobalt ion concentrations had similar correlations. Lymphocyte cuff thickness did not correlate with either total wear volume or whole blood metal ion concentrations, but correlated with the grade of necrosis.

**Conclusions:**

Bearing wear volume correlated with blood metal ion levels and the degree of necrosis and macrophage infiltration in periprosthetic tissues suggesting a dose-response relationship. Whole blood metal ion levels are a useful tool for clinician to estimate bearing wear and subsequent tissue response.

## Background

Adverse reaction to metal debris (ARMD) is still a major reason for revision surgeries in patients with metal-on-metal (MoM) hip implants. Although the use of MoM hip implants has been widely ceased, more than one million patients have received such a device [1] and those that have not been revised still pose an increased risk for implant failure. ARMD is an umbrella term describing a wide range of alterations seen macro- and microscopically in the periprosthetic tissue such as metallosis, necrosis, inflammation of different types and soft-tissue inflammatory lesions referred as pseudotumors [2–4].

Retrieval studies have investigated implant wear and its association to ARMD. Results of these studies have been inconclusive as adverse reactions have been observed both in patients with high and low wearing hip implants [5–11]. In their recent systematic review, Campbell et al. concluded that no clear dose-response relationship between wear and ARMD could be established due to the heterogeneity of the findings in the included studies. Studies that have investigated wear or indirect markers of wear, such as synovial fluid (SF) or whole blood (WB) metal ion concentrations, and the histopathological features of ARMD have also yielded inconsistent results [8, 9, 12–19]. Extra-articular tissues retrieved from patients with ARMD vary considerably in their histologic presentation. Most often tissues display prominent macrophage infiltration as a response to the cytotoxic metal wear debris with a variable amount of lymphocytic infiltration, either diffuse or aggregated [2, 3, 8, 13]. However, in a minority of patients with ARMD, there is heavy lymphocytic infiltration, resembling type IV hypersensitivity reaction [17, 20–23]. The presence of lymphocytes is usually accompanied with the presence of necrosis and this type of tissue response was first termed ALVAL (Aseptic Lymphocytic Vasculitis-Associated Lesion) by Willert et al. [20]. Terms ALVAL and ARMD have however been inappropriately used as synonyms in the recent literature [24]. Low bearing wear has been associated with a suspected metal hypersensitivity response in some studies [8, 9, 16]. Vice versa, high bearing wear has been associated with a macrophage-dominated foreign-body response. [8, 13]. In addition, low WB metal ion levels have been associated with lymphocyte-dominated tissue response and high metal ion levels with macrophage-dominated response [17]. Based on these findings, metal hypersensitivity to implant-derived debris has been hypothesized as a cause of ARMD in patients with low-wearing hip implants, and cytotoxic, macrophage dominated response in patients with high-wearing hip implants [8, 13, 16, 17] However, findings not supporting these hypotheses have been published as well [10, 12, 15, 18, 19].

The histopathology of ARMD has been well described but the literature regarding its association to implant wear is inconsistent. It is important to understand the true nature of the association between wear and histopathological findings in ARMD. After all, it is the histopathological changes – tissue destruction and inflammation – that lead to failure of MoM hip implants. Implant wear cannot be measured in-vivo and thus cannot be used in clinical decision making but there are reliable indirect measures of wear, such as WB metal ion levels, that are commonly used in the follow-up of patients with MoM hip replacements. To gain a better understanding of the relationships between histopathological findings, bearing wear and clinical markers of wear we aimed to investigate bearing surface wear volume, WB and SF metal ion concentrations as clinical markers of wear, and their associations with histopathological findings of the periprosthetic tissue in patients with Articular Surface Replacement (ASR) hip resurfacing device revised due to ARMD. Based on the previous literature we hypothesized that 1) low implant wear is associated with high amount of lymphocytes characteristic of an ALVAL response and 2) high implant wear is associated with high amount of macrophages characteristic of a foreign-body response to metal wear debris.

## Methods

Between the recall of the ASR MoM hip system (Depuy Orthopaedics, Warsaw, IN, USA) in August 2010 and the end of our recruitment period in January 2016, 114 ASR hip resurfacing devices in 107 patients have been revised at our institution. All consecutively revised patients who gave informed consent and fulfilled the following criteria were included in our study: 1) Revision was due to ARMD, 2) Retrieved components were available for bearing wear analysis and 3) Periprosthetic tissue sample was available for histopathologic analysis. After exclusion, 85 hips in 78 patients were included in our study. Twenty-one of these patients were referred to our institution from central hospitals from other hospital districts and 57 patients had had their index operation (primary arthroplasty) and follow-up at our institution. Surgery was performed by or under the direct supervision of 14 senior orthopaedic surgeons. The study was approved by the ethical committee of Pirkanmaa Hospital District (R11006).

Revision surgery was considered if 1) a clear pseudotumour (Imperial class 2A,2B or 3) [25] was observed on cross-sectional imaging regardless of symptoms or WB metal ion levels; or 2) the patient had elevated WB metal ion levels and hip symptoms despite normal findings in cross-sectional imaging; or 3) the patient had a continuously symptomatic hip or progressive symptoms regardless of imaging findings or metal ion levels. Symptoms included hip pain, discomfort, sense of instability, and/or impaired function of the hip and sounds from the hip (clacking, squeaking). WB metal ion levels were regarded as being elevated if either chromium or cobalt exceeded 5 ppb. Postoperatively, failure was classified as being due to ARMD on the basis of the following criteria [26, 27]: 1) there was presence of metallosis or macroscopic synovitis in the joint; and/or 2) a pseudotumor was found during revision; and/or 3) a moderate to high number of perivascular lymphocytes along with tissue necrosis and/or fibrin deposition was seen in the histopathologic sample; and 4) perioperatively there was no evidence of component loosening or periprosthetic fracture. In addition, infection was ruled out by obtaining multiple (at least five) bacterial cultures during revision surgery.

### Bearing wear analysis

The volume of material loss from the cup and head bearing surfaces was measured using a Zeiss Prismo (Carl Zeiss Ltd., Rugby, UK) coordinate measuring machine (CMM). A total of 400 polar scan lines on each surface were defined and up to 30,000 data points captured using a 2 mm ruby stylus; protocols for this method have been previously published [28]. An iterative least square fitting method was used to analyse the raw data captured by the CMM and the unworn geometry of the bearing surface was used to map regions of material loss from which the total volumetric loss was calculated. Wear rate (mm3/year) was further calculated by dividing total wear volume in cubic millimeters by implantation time in years.

### Histopathological analysis of the periprosthetic tissue

During every hip revision, a sample of the inflamed synovia or pseudotumor was obtained. For histopathological analysis, each tissue sample was formalin fixed. Several 10 μm microtome sections were made and embedded in paraffin. Standard hematoxylin and eosin staining was used. The sections were examined histologically under normal light with a Nikon Eclipse 50i (Nikon Corporation, Shinagawa, Tokyo, Japan). The samples were graded by a senior musculoskeletal pathologist (JP) using scoring principles adopted from the study by Natu et al. [2] (termed Natu grading in our study) and the ALVAL score previously described by Campbell et al. [8].

The Natu grading consisted of following parameters: 1) lymphocyte cuff thickness, 2) whether lymphocytic infiltrate was diffuse or aggregated, 3) presence of germinal centers, 4) histiocyte sheet thickness, 5) metal particle load within histiocytes, 6) extent of tissue necrosis, 7) presence of plasma cells and 8) presence of granulomas. Lymphocyte cuff thickness was calculated using a graticule. An average of five measurements was taken and graded as 0–3 (absent, 0.25 mm, 0.25–0.75 mm, >0.75 mm). Thickness of histiocyte sheets was also calculated using a graticule and graded 0–3 (absent, <1 mm, 1–2 mm, >2 mm). Metal particle load within histiocytes was graded as 0–4 as done in the assessment of iron decomposition in liver cells [29, 30]. The extent of overall tissue necrosis in a sample was graded based on the surface necrosis typing according to Davies et al. [22]. Type 1 surface contains intact synovial epithelium. Type 2 surface shows loss of synovial epithelial cells without fibrin deposition. In type 3 surface there is fibrin deposition and in type 4 surface there is extensive necrosis and loss of architecture. The extent of type 4 surface necrosis was used to grade the overall tissue necrosis in a given sample, as described by Natu et al. [2]. In grade 4 necrosis, more than 75% of the tissue sample showed type 4 surface necrosis. In grade 3 necrosis, between 25 and 75% showed type 4 surface necrosis. In grade 2 necrosis either less than 25% of the tissue showed type 4 surface necrosis or the tissue showed type 3 surface. In grade 1 necrosis, the sample consisted of type 2 surface.

ALVAL scoring consists of three subscores: synovial lining (0-3p), tissue organization (0-3p) and inflammatory infiltrate (0-4p). Both synovial lining and tissue organization reflect the degree of necrosis and higher scores mean higher degree of necrosis. Inflammatory infiltrate score reflects the predominant inflammatory cell type on a spectrum: 0 points means minimal infiltrates, 1p means predominantly macrophages, 2p means both macrophages and diffuse/perivascular lymphocytes, 3p means mostly lymphocytes in aggregates and some macrophages and 4p means large lymphocyte aggregates and little to no macrophages.

### Whole blood and synovial fluid metal analysis

Since January 2012, WB metal ion (Co and Cr) concentrations have been routinely measured as a part of the systematic follow-up program for patients with MoM hip replacements at our institution. All patients underwent WB analysis of Co/Cr following sampling from the antecubital vein using a 21-gauge needle connected to a Vacutainer system (Becton, Dickinson and Company, Franklin Lakes, NJ, USA) and trace-element blood tubes containing sodium ethylenediaminetetraacetic acid (EDTA). Standard operating procedures were established at the Finnish Institute for Occupational Health for Co and Cr measurement using dynamic reaction cell inductively coupled plasma (quadripole) mass spectrometry (Agilent 7500 cx, Agilent Technologies, Santa Clara, CA, USA). The laboratory technicians were blinded to all clinical outcomes. The samples were preserved in +6 °C to +8 °C prior to analysis.

Since October 2011, our MoM hip revision protocol has involved perioperative SF aspiration, which is always taken before opening the deep fascia using a standard 18- to 20-gauge needle connected to a Vacutainer system (Becton, Dickinson and Company, Franklin Lakes, New Jersey) and trace element tubes containing sodium EDTA. Similar procedures were used for SF metal ion concentration measurement as described above for WB.

### Statistical methods

Spearman rank correlation was used to study the associations between wear volume, WB and SF metal ion concentrations, and histopathological findings due to non-normal distribution of these variables. Medians were calculated for wear volume, WB and SF metal ion concentrations. To compare these median values between different subgroups, nonparametric Mann-Whitney U-test was used. When analyzing the correlation between WB metal ion concentrations and other factors, we only included patients with unilateral hip arthroplasties (57 hips) to avoid the confounding effect of metal ions being released to the blood from the other implant. The threshold for statistical significance was set to 0.05. The analyses were conducted using IBM SPSS software (IBM Corp. Released 2012. IBM SPSS Statistics for Windows, Version 21.0. Armonk, NY: IBM Corp.).

## Results

Of the 85 hips included in the study, 56 were explanted from female patients and 29 from male patients. Mean age at the time of the revision surgery was 57.3 years (SD 10.3 years). Mean follow-up time between index operation and revision surgery was 5.4 years (SD 1.8 years).

Volumetric wear analysis of the explanted components demonstrated a wide range of wear in both the acetabular cup and femoral head (Table 1). Wear rates were also highly variable with a median of 9.0 mm3/year (range 1.1…99.7 mm3/year). In a vast majority of the components (85.1%), the femoral head was more worn than the acetabular cup. Median ratio for head wear to cup wear was 1.7 (range, 0.5…10). In addition to actual volumetric component wear, also WB and SF metal ion levels, serving as indirect markers of wear, were highly variable (Table 2). The total wear volume of the head and cup strongly correlated with WB metal ion concentrations (Cr: ρ = 0.80, *p* < 0.001 and Co: 0.84, *p* < 0.001) and moderately with SF metal ion concentrations (Cr: ρ = 0.50, *p* < 0.01 and Co: ρ = 0.41, *p* = 0.027). Wear rate had slightly stronger correlation with WB metal ion concentrations (Cr: ρ = 0.87, *p* < 0.001 and Co: 0.89, *p* < 0.001) and SF metal ion concentrations (Cr: ρ = 0.71, *p* < 0.001 and Co: 0.66, *p* < 0.01) than total wear volume.

**Table 1:** Median volumetric wear and range for acetabular and femoral components and both combined

Component	Median volumetric wear (mm^3^)	Range (mm^3^)
Acetabular cup	14	2–247
Femoral head	24	4–485
Both combined	39	7–541

**Table 2:** Median concentrations (μg/l) and ranges (μg/l) for chromium and cobalt ions in both whole blood and synovial fluid

Metal ion	Whole blood	Range	Synovial fluid	Range
Chromium	9.7	0.5–93.9	701	7.0–52360
Cobalt	15.4	0.7–224.7	281.5	27.0–14870

**Fig. 1:**
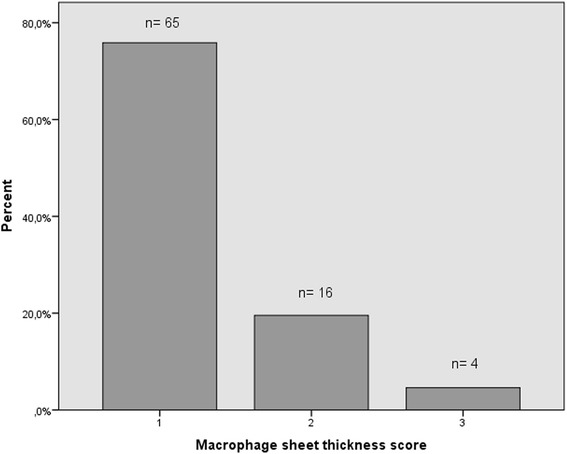
Distribution of macrophage sheet thickness scores among all periprosthetic tissue samples

**Fig. 2:**
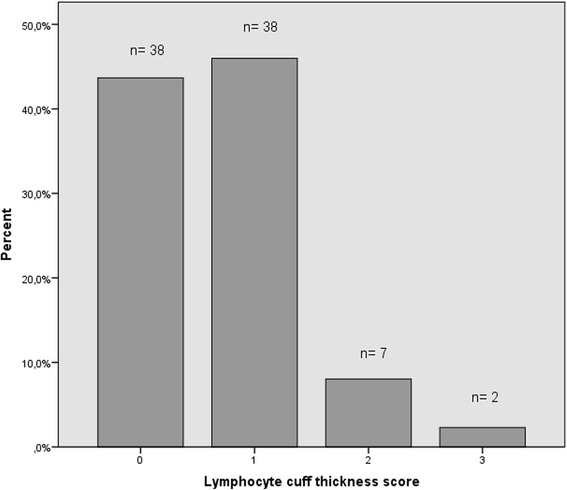
Distribution of lymphocyte cuff thickness scores among all periprosthetic tissue samples

Histologically, variable amounts of macrophages, lymphocytes and necrosis were seen in the tissue samples. One or more germinal centers were present in 5 samples (5.7% of all samples). One or more granulomas were present in 14 samples (16.5% of all samples). All tissue samples evinced at least some degree of macrophage infiltration (macrophage sheet thickness score of at least 1) and in most cases it was low to moderate (Fig. 1). In regard to lymphocyte infiltration, most tissues evinced little to no lymphocytes and in only a minority of the samples the infiltrate was prominent (Fig. 2). All cases with heavy lymphocyte infiltration (scores 2 or 3) had a macrophage sheet thickness score of 1, ie. there was only little macrophage infiltration in these tissues. Eight of the nine tissue samples with heavy lymphocyte infiltration had grade 4 necrosis and the ninth had grade 3 necrosis. Median wear rate for these tissues was higher than that for the tissues with lower numbers of lymphocytes (Table 3). Lymphocyte cuff thickness correlated positively with the grade of necrosis (ρ = 0.41, *p* < 0.001) and inflammatory infiltrate score (ρ = 0.79, *p* < 0.001).. All five tissue samples with germinal centers had grade 4 necrosis. The wear volume or wear rate of these cases did not differ from cases without germinal centers (median wear volume in cubic millimeters 61 versus 37.5, *p* = 0.94; median wear rate in cubic millimeters/year 11.9 versus 8.8, *p* = 0.86). Tissues with one or more granulomas were associated with higher total wear volume and wear rate when compared to tissues with no granulomas (Table 4). Grade of necrosis correlated positively with synovial lining score (ρ = 0.86, *p* < 0.001) and tissue organization score (ρ = 0.80, *p* < 0.001).

**Table 3:** Wear rates according to lymphocyte cuff thickness

	Lymphocyte cuff <2	Lymphocyte cuff 2 or 3	*P*-value
Median wear rate (mm3/year)	8.1	14.5	0.054
Range (mm3/year)	1.1 … 99.8	3.7 … 48.0

**Table 4:** Wear volume and rate: comparison between patients with one or more granulomas and those without granulomas

	Granuloma present	Granuloma absent	*P*-value
Median total wear volume (mm3)	106.5	31.0	0.016
Range (mm3)	10…378	7…541	
Median wear rate (mm3/year)	16.3	8.1	0.035
Range (mm3/year)	1.6…99.8	1.1…86.4	

**Fig. 3:**
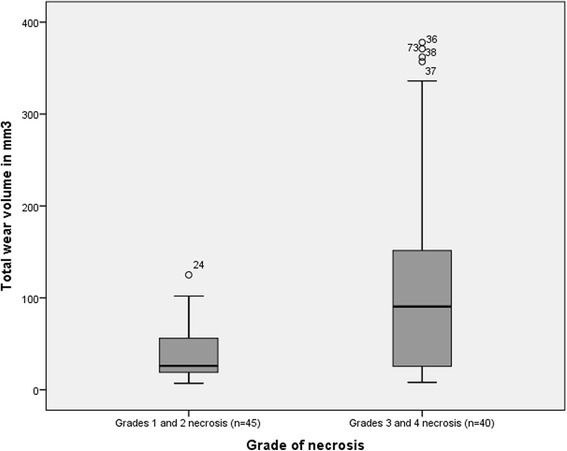
The difference in median total wear volume (head and cup) in patients with low-grade necrosis (grades 1 and 2) versus patients with high-grade necrosis (grades 3 and 4), *p* < 0,001

Correlations between histological variables, total wear volume of the head and cup components, wear rate as well as WB and SF metal ion concentrations are listed in Table 5. Total wear volume correlated with macrophage sheet thickness, grade of necrosis (Fig. 3), synovial lining score, tissue organization score and total ALVAL score. Wear rate had similar correlations but the correlation with macrophages did not quite reach statistical significance. WB cobalt and chromium ion concentrations had similar correlations. SF chromium ion concentration correlated with grade of necrosis, synovial lining score and total ALVAL score. SF cobalt ion concentration correlated with all but macrophage sheet thickness. Neither wear volume, wear rate, WB metal ion concentrations or SF chromium ion concentration were associated with lymphocyte cuff thickness or presence of germinal centers. However, SF cobalt ion concentration did correlate with lymphocyte cuff thickness.

**Table 5:** Spearman rho correlation coefficients and associated *p*-values for correlations between total wear volume, wear rate, indirect markers of wear (whole blood and synovial fluid metal ion concentrations) and histopathological grading (Natu and ALVAL)

	Natu grading			ALVAL grading			
	Lymphocytic cuffing	Macrophage sheet thickness	Grade of necrosis	Inflammatory infiltrate score	Synovial lining score	Tissue organization score	Total ALVAL score
Total wear volume	rho = 0.11 *p* = 0.32	rho = 0.25* *p* = 0.020	rho = 0.35* *p* < 0.01	rho = 0.13 *p* = 0.23	rho = 0.37* *p* < 0.01	rho = 0.25* *p* = 0.023	rho = 0.31* *p* < 0.01
Wear rate	rho = 0.17 *p* = 0.12	rho = 0.20 *p* = 0.069	rho = 0.42* *p* < 0.0001	rho = 0.16 *p* = 0.15	rho = 0.48* *p* < 0.0001	rho = 0.35* *p* < 0.01	rho = 0.40* *p* < 0.001
WB Cr	rho = 0.089 *p* = 0.51	rho = 0.30* *p* = 0.024	rho = 0.45* *p* < 0.001	rho = 0.19 *p* = 0.26	rho = 0.54* *p* < 0.001	rho = 0.33* *p* = 0.015	rho = 0.48* *p* < 0.001
WB Co	rho = 0.18 *p* = 0.18	rho = 0.29* *p* = 0.029	rho = 0.51* *p* < 0.001	rho = 0.26 *p* = 0.055	rho = 0.60* *p* < 0.001	rho = 0.40* *p* < 0.01	rho = 0.55* *p* < 0.001
SF Cr	rho = 0.30 *p* = 0.12	rho = 0.16 *p* = 0.40	rho = 0.48* *p* < 0.01	rho = 0.25 *p* = 0.19	rho = 0.56* *p* < 0.01	rho = 0.34 *p* = 0.070	rho = 0.53* *p* < 0.01
SF Co	rho = 0.49* *p* < 0.01	rho = 0.17 *p* = 0.37	rho = 0.54* *p* < 0.01	rho = 0.44* *p* = 0.017	rho = 0.47* *p* = 0.011	rho = 0.38* *p* = 0.045	rho = 0.57* *p* < 0.01

Values that are statistically significant are flagged with *

## Discussion

In the present study, a spectrum of inflammatory and necrotic changes associated with ARMD were seen – variable macrophage and lymphocyte infiltration and necrosis in periprosthetic tissues [2–4, 8, 21]. Most patients evinced low-to-moderate macrophage infiltration and little to no lymphocyte infiltration. A few patients evinced a very prominent lymphocyte infiltration with grade 4 necrosis typical of an ALVAL response first proposed by Willert et al. [20]. The thickness of lymphocyte cuffs correlated positively with the degree of necrosis. Bearing wear and WB metal ion concentrations correlated positively with the number of macrophages and the degree of necrosis.

Our study is not without limitations. Firstly, due to the periprosthetic tissue being sampled only at the time of revision surgery, it is difficult to say about the natural history of ARMD. Secondly, tissue samples were analyzed by one observer only. However, multiple microtome sections of each sample were made and analyzed by a senior musculoskeletal pathologist well acquainted with ARMD histopathology. Thirdly, we did not perform a priori sample size calculation. Our study was retrospective of nature and patients were included on an “all-comer” basis. However, a posteriori power analysis revealed that our study has 90% power (10% beta) to detect 0.35 correlation (medium effect size) with a type I error probability of 5% (alfa). Fourthly, although we consecutively recruited patients, not all patients who underwent surgery because of ARMD during the recruitment period were included due to refused consent, missing tissue samples and missing wear data on some patients. Thus, our patient series is not completely consecutive. However, the number of excluded patients was low in comparison to the number of consecutive patients included. Indeed, the large number of patients included is a major strength of our study. Another strength is that we only included patients revised for ARMD and with identical hip resurfacing implants. Thus, our data specifically describes patients with ARMD while minimizing the confounding effect from having different implant designs or failure modes other than ARMD. Also, there was no confounding effect from possible trunnion wear debris as in the case of THRs since we only investigated the effects of bearing wear debris.

Metal debris originating from the bearing surfaces and/or trunnion has been shown to have cytotoxic effects [31–34]. It has been suggested that the cytotoxicity of metal debris further leads to tissue destruction and macrophage recruitment to clear the tissue and metal debris [3, 21]. In support of this, we observed a correlation between implant wear and the number of macrophages as well as the degree of necrosis but not with the number of lymphocytes. Similar findings were made in a study by Grammatopoulos et al. [13]. Langton et al. however did not find correlation between wear and the amount of macrophages or necrosis [6]. We also observed that the presence of granulomas was associated with increased total wear volume. Granulomas are thought to form in response to high number of wear particles and our results support this idea [35]. High wear, or high WB metal ion concentrations, have been associated to macrophage-dominated tissue responses in other studies as well [8, 16, 17]. Metal hypersensitivity leading to type IV response with strong lymphocytic infiltration has been suggested as a cause of failure in those patients with low wear [8, 16, 17]. Contrary to our hypothesis, this was not observed in our study. In fact, patients with heavy lymphocytic infiltration had higher wear rate than patients with lower numbers of lymphocytes. These findings suggest that excessive metal debris accumulation was the cause of lymphocytic infiltration in these patients, not metal hypersensitivity. Grammatopoulos et al. also did not find correlation between wear and lymphocytic infiltration, but noted the presence of a patient subgroup with hypersensitivity-related histopathological findings and simultaneous low bearing wear, suggesting metal hypersensitivity as a cause of failure in those patients [13].

There are several reasons that likely contribute to the inconsistency of findings between different histopathological studies. It is possible and probable that some patients have both high-wearing implants and an underlying hypersensitivity response that would have evoked even in the presence of a low-wearing implant. This combination may result in a mixed-type tissue response that has the characteristics of both wear-related foreign-body response and hypersensitivity-related type IV tissue responses and therefore makes it difficult to distinguish between the two based on implant wear or WB metal ion levels alone. Also, threshold for the onset of adaptive immune response is likely variable between individuals [13]. This would explain why some patients tolerate extensive amount of wear debris and some patients develop ALVAL in the presence of a low wearing MoM hip replacement. In addition to patient susceptibility, differences in implant types among studies may play a role. Substantially higher failure rates have been reported for ASR XL THRs in comparison with ASR hip resurfacings [5, 19]. ASR XL and ASR have similar bearing couples, but in the ASR XL there is trunnion-interface between the titanium stem and CoCr head that serves as an additional source of metal debris. It has been shown that wear from the trunnion is different in nature compared to bearing surface wear and may lead to lymphocytic and necrotic tissue responses [14, 36, 37], possibly contributing to the inconsistencies between other recent histopathological studies.

Natu et al. suggested that the lymphocytic immune response in patients with MoM implants is a dynamic process, beginning with perivascular lymphocytic aggregates and leading to formation of lymphoid follicles with germinal centers, also termed as tertiary lymphoid organs (TLOs) [2]. TLOs are capable of forming new B and T cells locally. TLOs are seen in affected tissues of patients with chronic autoinflammatory diseases such as rheumatoid arthritis, Sjogrens syndrome and Hashimoto’s thyroiditis and are considered to be formed as a response to a persistent antigen that cannot be eliminated [38]. Mittal et al. demonstrated the presence of TLOs and associated chemokines in tissues of patients with failed MoM hip implants and suggested that these patients form a specific pathological subset [39] in addition to the well-established foreign-body response [3, 13] and ALVAL response [8, 20, 40]. In keeping with findings by Natu et al. and Mittal et al., we found that a minority of the patients displayed lymphoid follicles with germinal centers. Further, all tissue samples displaying germinal centers had grade 4 necrosis. This suggests that the formation of TLOs in periprosthetic tissues is associated with tissue destruction, possibly accelerating the process of implant failure. In line with this is a finding that the presence of TLOs has been associated with tissue destruction and loss of function in autoimmune diseases [38]. In the present study, we also observed that even in the absence of germinal center containing lymphoid follicles, the thickness of lymphocytic cuffs correlated with the grade of necrosis. Whether the lymphocytic immune response, also termed ALVAL, is a dynamic process leading to formation of TLOs as Natu et al. suggested [2] or whether patients with TLOs define their own distinct pathological subset as Mittal et al. suggested [39] requires further research. However, due to the cross-sectional nature of histopathological studies, it is difficult to investigate the natural history of ARMD.

ALVAL grading introduced by Campbell et al. [8] has been used in several studies [9, 16, 18, 41] but other grading systems have been used as well [2, 13, 21, 22, 36]. This makes comparison between studies difficult. In the present study, all tissue samples were analyzed according to two grading criteria: ALVAL grading and grading principles established by Natu et al. [2]. ALVAL grading is relatively restricted compared to the Natu grading as it only includes inflammatory infiltrate score, synovial lining score and tissue organization score. Moreover, as discussed by Ricciardi et al., both synovial lining score and tissue organization score reflect the degree of necrosis [21]. A strong correlation between these scores and the Natu score for necrosis was observed in our study. ALVAL score was originally designed to help distinguish failures related to high wear from failures related to suspected hypersensitivity (ALVAL) response. Although necrosis is often seen with ALVAL response, it is not specific for ALVAL as it is also seen with macrophage-dominated foreign body reactions with possible related cytotoxicity [3, 13]. This leaves only the inflammatory infiltrate score in ALVAL grading specific for ALVAL response. In the present study, a strong correlation between lymphocyte cuff thickness and inflammatory infiltrate score was observed. This indicates that the inflammatory infiltrate score is useful in distinguishing lymphocyte-dominated responses from those that are not lymphocyte-dominated. Phillips et al. also concluded that ALVAL scoring is useful for distinguishing between macrophage and lymphocyte responses [42]. Inflammatory infiltrate score involves evaluation of both lymphocytic and macrophagic components. However, both lymphocytes and macrophages are often seen in periprosthetic tissues. Grammatopoulos et al. suggested that an easier method to identify ALVAL responses from wear-related responses would be to measure only the thickness of lymphocytic cuffing, termed Oxford-ALVAL score in their study [13]. We agree with Grammatopoulos et al. and find that separate scores for evaluation of macrophage and lymphocyte infiltration provide more information about the failure mechanism and lead to easier comparison between histopathological studies.

**Fig. 4:**
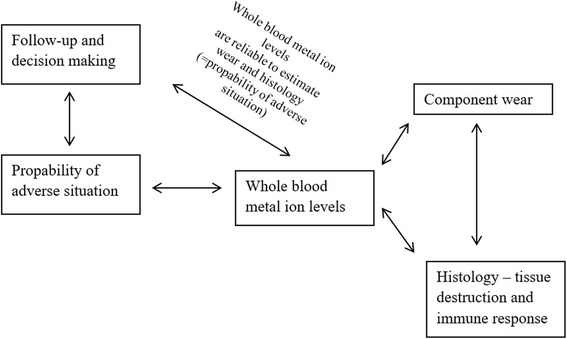
A schematic diagram for the relationships between wear, histology of the periprosthetic tissues, whole blood metal ion levels and follow-up of the patients

In the present study, WB metal ion levels had a strong correlation with bearing wear volume, wear rate and a moderate correlation with several histopathological features. SF metal ion levels also correlated with bearing wear volume, wear rate and some of the histopathological features, but the correlations were weaker. Wear rate had a stronger correlation with WB and SF metal ion levels than total bearing volume. Wear rate, WB and SF metal ion levels likely reflect the recent burden of metal debris whereas total wear volume reflects the amount of total wear accumulated during implantation time. In a study by De Smet et al. both WB and SF metal ion levels were found to correlate well with linear wear of the femoral component [43]. Langton et al. also noted a correlation between wear volume and WB metal ion levels [6]. Interestingly, WB metal ion levels had stronger correlation with histopathological findings compared to SF metal ion levels in the present study. This is supported by a recent study by Reito et al. who found that SF metal ion levels had relatively poor correlation with histopathological findings [12]. SF aspiration is an invasive procedure and the measurement does not seem to provide any additional information compared to WB measurement. Measurement of WB metal ion levels is a reliable, indirect way to gain information of the in-situ wear process, inflammation and tissue destruction. Clinicians should use WB metal ion levels in the follow-up of patients with MoM hip implants and closely monitor those patients with elevated metal ion levels. A schematic diagram for the relationships between wear, histology of the periprosthetic tissues, WB metal ion levels and follow-up of the patients is presented in Fig. 4.

## Conclusions

In the present study with failed ASR hip resurfacings, total wear volume, wear rate and WB metal ion concentrations correlated with the number of macrophages and the degree of necrosis, but not with the amount of lymphocytes. Most tissue samples evinced macrophages but little to no lymphocytes typical of a non-specific foreign-body response. A minority of the samples evinced strong lymphocyte infiltration combined with high amount of necrosis, typical of an ALVAL response. However, contrary to our hypothesis, this type of response was not associated with low implant wear in the present study. The significance of patient susceptibility in the development of ARMD is poorly understood and it is not currently known which factors lead to the adaptive lymphocytic response seen in some patients. Future studies should be directed to understand the pathophysiological mechanisms behind different types of tissue responses seen in patients with MoM hips. WB metal ion levels correlated with total wear volume, wear rate and histopathological findings. Measurement of WB metal ion levels is useful in the follow-up of patients with hip resurfacings as it provides information of the wear process, inflammatory response and tissue destruction.

## Abbreviations

ALVAL: Aseptic lymphocytic vasculitis-associated lesion
ARMD: Adverse reaction to metal debris
ASR: Articular surface replacement
CoC: Ceramic-on-ceramic
MoM: Metal-on-metal
MoP: Metal-on-polyethylene
SF: Synovial fluid
THR: Total hip replacement
TLO: Tertiary lymphoid organ
WB: Whole blood
